# Temporal changes in innate immune signals in a rat model of alcohol withdrawal in emotional and cardiorespiratory homeostatic nuclei

**DOI:** 10.1186/1742-2094-9-97

**Published:** 2012-05-24

**Authors:** Kate Freeman, Anthony Brureau, Rajanikanth Vadigepalli, Mary M Staehle, Melanie M Brureau, Gregory E Gonye, Jan B Hoek, D Craig Hooper, James S Schwaber

**Affiliations:** 1 Daniel Baugh Institute for Functional Genomics and Computational Biology, Department of Pathology, Anatomy and Cell Biology, Thomas Jefferson University, 1020 Locust Street, Philadelphia, PA, 19107, USA; 2 Department of Chemical Engineering, Rowan University, 201 Mullica Hill Road, Glassboro, NJ, 08028, USA; 3 Department of Cancer Biology and Neurosurgery, Thomas Jefferson University, 111 South 11th Street, Philadelphia, PA, 19107, USA

**Keywords:** Alcohol, Withdrawal, Inflammation, TNF-alpha, MCP-1, Gene expression, IHC

## Abstract

****Background**:**

Chronic alcohol use changes the brain’s inflammatory state. However, there is little work examining the progression of the cytokine response during alcohol withdrawal, a period of profound autonomic and emotional upset. This study examines the inflammatory response in the central nucleus of the amygdala (CeA) and dorsal vagal complex (DVC), brain regions neuroanatomically associated with affective and cardiorespiratory regulation in an *in vivo* rat model of withdrawal following a single chronic exposure.

****Methods**:**

For qRT-PCR studies, we measured the expression of *TNF-α*, *NOS-2*, *Ccl2 (MCP-1)*, MHC II invariant chain *CD74*, and the TNF receptor *Tnfrsf1a* in CeA and DVC samples from adult male rats exposed to a liquid alcohol diet for thirty-five days and in similarly treated animals at four hours and forty-eight hours following alcohol withdrawal. ANOVA was used to identify statistically significant treatment effects. Immunohistochemistry (IHC) and confocal microscopy were performed in a second set of animals during chronic alcohol exposure and subsequent 48-hour withdrawal.

****Results**:**

Following a chronic alcohol exposure, withdrawal resulted in a statistically significant increase in the expression of mRNAs specific for innate immune markers *Ccl2*, *TNF-α*, *NOS-2*, *Tnfrsf1a*, and *CD74*. This response was present in both the CeA and DVC and most prominent at 48 hours. Confocal IHC of samples taken 48 hours into withdrawal demonstrate the presence of TNF-α staining surrounding cells expressing the neural marker NeuN and endothelial cells colabeled with ICAM-1 (CD54) and RECA-1, markers associated with an inflammatory response. Again, findings were consistent in both brain regions.

****Conclusions**:**

This study demonstrates the rapid induction of *Ccl2*, *TNF-α*, *NOS-2*, *Tnfrsf1a* and *CD74* expression during alcohol withdrawal in both the CeA and DVC. IHC dual labeling showed an increase in TNF-α surrounding neurons and ICAM-1 on vascular endothelial cells 48 hours into withdrawal, confirming the inflammatory response at the protein level. These findings suggest that an abrupt cessation of alcohol intake leads to an acute central nervous system (CNS) inflammatory response in these regions that regulate autonomic and emotional state.

## **Background**

The relationship between alcohol use, withdrawal and brain inflammation is complex and of considerable interest in light of the high incidence of alcohol use and misuse and the potential impact of alcohol-mediated brain inflammation on neurologic and emotional health [1]. Recent evidence suggests that chronic alcohol exposure alters neuroimmune function, creating a proinflammatory state [2]. For example, astrocytes isolated from the cerebral cortex of alcohol-exposed rats show increased inductible nitric oxide synthase (NOS-2), cyclooxygenase-2, (COX-2) and IL-1β levels in culture [3]. Moreover, 10-day intragastic alcohol exposure amplifies and prolongs the brain’s production of TNF-α, monocyte chemotactic protein-1 (MCP-1) and IL-1β in response to a peripheral lipopolysaccharide (LPS) injection in rats [4]. The possibility that alcohol could have direct effects on central nervous system (CNS)-resident cells is raised by the finding that alcohol causes a dose- and time-dependent induction of TNF-α and Il-1β in cultured microglia [5]. Studies of postmortem brain samples suggest that these findings are relevant to human neuropathology. Histologic changes in microglia, increases in MCP-1 mRNA levels [6], and altered transcriptional regulation of NF-kB [7] have been identified in brain tissue from alcoholics.

Together, these studies suggest that alcohol exposure affects innate immune system function. Normally, the innate immune system protects vertebrates from infection by reacting to nonspecific signals of infection, cellular stress and injury. If such signals, like abnormal lipids, reactive oxygen or nitrogen species, nucleic acids or other cellular debris are encountered, cells of the innate immune system activate various toll-like receptors, downstream JAK-Stat and MAPK signaling pathways [8], biosynthetic processes [9] and gene expression [10,11], that promote a proinflammatory state. This response activates within minutes to hours and involves the production of various chemokines, cytokines and angiogenic factors like TNF-α and interleukins as well as nonprotein signals, such as nitric oxide, that are synthesized by resident and recruitable cells [8]. In the central nervous system, innate immune functions are principally carried out by microglia [12,13], although astrocytes [14], endothelial cells [15], neurons [16,17] and in pathological conditions, infiltrating peripheral white blood cells [18,19] may all contribute. As a result of this intercellular cytokine-mediated paracrine and autocrine signaling, the innate response is activated, propagated and amplified, providing both immune defense and tissue repair [2].

There is accumulating evidence that innate neuroinflammatory processes with pathological properties are activated by chronic alcohol exposure [2,20-22]. Yet, how these processes are engaged during withdrawal and their relationship to the resultant anxiety and cardiorespiratory disturbance are poorly understood. The hallmarks of alcohol withdrawal are potentially life-threatening emotional and autonomic instability characterized by anxiety, agitation, delirium, and sympathetic signs of elevated heart rate and blood pressure [23-27]. These disturbances are anatomically associated with the central nucleus of the amygdala (CeA) and the dorsal vagal complex (DVC), two key viscerosensory nuclei. The former is part of the limbic system and known to be altered in alcohol exposure and withdrawal [28-30]; The latter is a brainstem nucleus that receives afferent from the viscera and moderates vagal influence on cardiac and respiratory functions, also profoundly affected by withdrawal [31]. The CeA receives direct autonomic input from the DVC [32,33], and together they act as part of a viscerosensory and motor circuit to integrate and modulate physical and emotional aspects of autonomic outflow, motivation, affect and emotional learning. Here, we examine the CeA and DVC, specific neural structures associated with the primary symptoms of withdrawal, for evidence of an altered inflammatory state following cessation of the chronic alcohol diet.

It may be expected that removal of alcohol should promote resolution of any proinflammatory state resulting from chronic exposure, leading to recovery. However, there is evidence to suggest that the opposite may occur; repeated cycles of alcohol exposure and withdrawal may exacerbate cellular level oxidative stress and inflammation [34,35]. Abrupt changes in the extracellular CNS environment as alcohol levels decrease may directly stress cells, particularly after prolonged exposures leading to molecular adaptation and dependence. By surveying important features of innate immunity during the first 48 hours of alcohol withdrawal in rats following a single period of chronic exposure, here we differentiate between these two possible outcomes. In the CeA, changes consistent with an increased proinflammatory response were identified. Additionally, we identified similar changes in the DVC, suggesting that inflammatory signals are present in these regions anatomically associated with emotional and autonomic instability during early withdrawal.

## **Methods**

### **Animals**

Male Sprague Dawley rats (>120 g, Harlan, Indianapolis, IN, USA) were housed individually in the Thomas Jefferson University (TJU) Alcohol Research Center Animal Core Facility. Facilities were maintained at constant temperature and humidity with 12/12 hour light cycles. For quantitative reverse transcription polymerase chain reaction (qRT-PCR) studies, animals were assigned to four treatment groups: control, chronic alcohol exposure, four-hour withdrawal or forty-eight-hour withdrawal following a thirty-five-day chronic exposure. In immunohistochemistry studies, animals were assigned to three treatment groups: control, chronic alcohol exposure, or 48-hour withdrawal following chronic exposure. In this set of experiments, for technical reasons, animals were used that had received eight months of ethanol or control diet. As in previously published studies from this facility [31] and elsewhere [25], chronic and withdrawal animals were fed the Lieber-DeCarli liquid alcohol diet (36 % of calories as alcohol) *ad libitum* throughout the exposure period [36,37]. Control rats were fed a liquid diet where alcohol was isocalorically replaced with carbohydrate and diet volume equaled the average consumption of alcohol-fed littermates. No additional water or chow was provided to any of the animals during the study period, ensuring that the animals’ entire nutrient and fluid intake came from the alcohol diet. To initiate withdrawal, the alcohol diet was removed at the appropriate time to ensure that all animals would be sacrificed five hours into the light cycle, to account for potential differences due to diurnal factors. All protocols were approved by the TJU Institutional Animal Care and Use Committee.

In the Lieber-DeCarli protocol, blood alcohol levels are not externally controlled during the experiment. Rather, each animal is allowed to self-regulate its oral alcohol intake. Studies using the Lieber-DeCarli method in this facility and elsewhere have shown peak blood alcohol concentrations of 20 to 30 mM with an average daily alcohol intake of 12 to 16 g/kg in rats following long-term exposure (>3 weeks) [38-40]. Rats on the full-strength liquid alcohol diet in our facility have comparable intake, as previously published [31]. There were no differences in average intake between the chronic alcohol-exposed and withdrawn animals.

**Figure 1:**
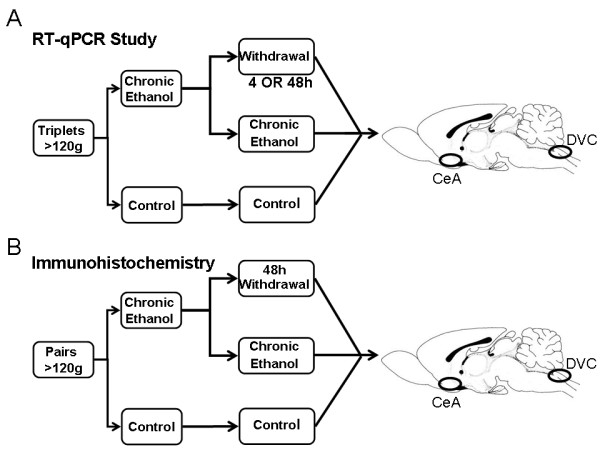
**Experimental Design.(A)** For the gene expression qRT-PCR study, triplet rats were assigned to control, chronic exposure, and four- or forty-eight-hour withdrawal. Following a 35-day liquid alcohol exposure, animals were sacrificed and the RNA extracted from their CeA and DVC. **(B)** For IHC study, four pairs of animals were assigned to either the chronic exposure or 48 hours of alcohol withdrawal following eight months on the alcohol diet. Forty-eight hours prior to the time of sacrifice, the withdrawal animals had their diets removed to initiate withdrawal. Following intracardiac perfusion, both rats were sacrificed and their CeA and DVC collected for immunohistochemistry and confocal microscopy. CeA, central nucleus of the amygdala; DVC, dorsal vagal complex; qRT-PCR, quantitative reverse transcription polymerase chain reaction; IHC, immunohistochemistry.

To initiate withdrawal, the alcohol diet was replaced with the control diet. Matched chronically exposed rats were given free access to the alcohol diet until sacrifice. Previous studies and our experience show that symptomatic alcohol withdrawal in rats following a long-term liquid alcohol diet begins within hours and resolves over a two- to three-day period [41-44], though withdrawal symptomatology was not systematically assessed during this study. Studies of alcohol clearance following the cessation of the liquid alcohol diet have shown that clearance rate is approximately linear, and is reduced to less than 25 % of original levels seven hours after removal of the alcohol diet [39]. Similarly, liquid ethanol diet exposures longer than 10 days generate physiologic and behavioral dependence, as evidenced by autonomic and somatic dysfunction upon withdrawal. Within withdrawal’s first four hours, these animals show an increased susceptibility to audiogenic convulsions, fragmented sleep, piloerection, tail stiffening, reduced grooming, abnormal gait, reduced motor activity, exaggerated startle, vocalizations and tremors [40,42] that resolve over the first 48 to 72 hours [40,43]. While the behavioral and electrographic abnormalities associated with withdrawal from the Lieber-DeCarli diet over time are well characterized, there is little information available about molecular changes in the inflammatory state of the brain during this period. To examine these changes in the central nervous system inflammatory response during this period, we sampled following chronic exposure and at four and forty-eight hours after alcohol removal. Figure 1 shows a schematic of the experimental design.

### **CeA and DVC microdissection and qRT-PCR**

At the assigned time of sacrifice (four or forty-eight hours after removal of the alcohol diet for withdrawal animals), withdrawn, chronically alcohol-exposed, and match-fed control animals were sacrificed by rapid decapitation and brainstems were excised, placed into ice-cold artificial cerebral spinal fluid (ACSF: 10 mM HEPES, pH 7.4; 140 mM NaCl; 5 mM KCl; 1 mM MgCl_2_; 1 mM CaCl_2_; 24 mM D-glucose) and secured with agarose for sectioning (4 % UltraPure™ low melting point agarose (Invitrogen, Carlsbad, CA, USA) in ACSF). Transverse sections were made with a McIlwain tissue chopper (McIlwain, Gamshall, England) for CeA (625 μm) and DVC (275 μm) microdissection with size-matched micropunches (≤1.25 mm; Stoelting, Wood Dale, IL, USA), as previously reported [45]. Bilateral region punches from one animal were treated as a single sample. Total sample numbers were as follows: CeA Control N = 10, chronic ethanol exposure N = 5, 4-hour withdrawal N = 5, 48-hour withdrawal N = 3; NTS Control =11, chronic N = 5, 4-hour withdrawal N = 7, 48-hour withdrawal N = 5.

Total RNA was extracted with either the RNeasy or the AllPrep DNA/RNA extraction kit (Qiagen, Valencia, CA, USA), DNAase treated (DNA-free RNA kit, Zymo Research, Orange, CA, USA), and stored at −80°C. Concentration and integrity were assessed with an ND-1000 (NanoDrop, Wilmington, DE, USA) and RNA Nano 6000 chips on an Agilent 2100 Bioanalyzer. cDNA was reverse transcribed with SuperScript II (Invitrogen) from 100 ng total RNA and stored at −20°C.

Intron-spanning PCR primers and probes were designed using the Roche Universal Probe Library Assay Design Center (http://www.universalprobelibrary.com) as indicated: TNF-α forward gtagcccacgtcgtagcaa reverse ggttgtctttgagatccatgc and UPL Probe #79, Ccl2 (MCP-1) forward agcatccacgtgctgtctc reverse gatcatcttgccagtgaatgag and UPL Probe #62, NOS-2 forward ggtctttgaaatccctcctga reverse agctcctggaaccactcgta and UPL Probe #67, the TNF receptor Tnfrsf1a forward aatgggggagtgagagagg reverse acccctgatgggtgtatcc and UPL Probe #21, and the MHC II invariant chain CD74 forward cttccatgtccagtggctct reverse gctgttgtttgaaatgagcaag and UPL Probe #65. The standard BioMark™ protocol was used to preamplify cDNA samples for 16 cycles using TaqMan PreAmp Master Mix per the manufacturer’s protocol (Applied Biosystems, Foster City, CA, USA). qPCR reactions were performed using BioMark™ 96.96 Dynamic Arrays (Fluidigm, South San Francisco, CA, USA) enabling quantitative measurement of multiple mRNAs and samples under identical reaction conditions [46,47]. Runs were forty cycles (fifteen seconds at 95°C, five seconds at 70°C, sixty seconds at 60°C). C_T_ values were calculated by the Real-Time PCR Analysis software (Fluidigm) and software-designated failed reactions were discarded from the analysis.

### **qRT-PCR data normalization and analysis**

Normalization (∆C_T_) to the average expression of the housekeeping genes *Actb* and *Rpl13a* was performed using the R statistical computing package [48-50]. Two-way analysis of variance (ANOVA) performed on ∆C_T_ values was used to identify genes with a significant region or treatment effect. ANOVA identifies the variability in data that is associated with and potentially attributable to each experimental factor (treatment and region). Post hoc significance testing was performed using a Tukey’s honestly significant difference (HSD) test to identify pair-wise differences between treatment times. Here, a region ANOVA *P* value of less than 0.05 indicates a significant difference in mRNA levels in the CeA and DVC, while a treatment ANOVA *P* value of less than 0.05 indicates a significant cumulative effect on mRNA levels over the four treatment conditions: control, chronic alcohol exposure and four-hour and forty-eight-hour withdrawal. Tukey’s HSD values of less than 0.05 identify pair-wise differences between individual treatment conditions. Mean differences in mRNA expression between treatment groups are expressed as ∆∆C_T_ values by calculating the average ∆C_T_ values of each gene at a given treatment time and subtracting this value from the mean ∆C_T_ of all controls [51]. In this way, a ΔΔCT=1 corresponds to a 2‐fold increase in expression. All statistical tests were conducted at a 95 % confidence level (*P* ≤ 0.05).

### **Intracardiac perfusion, immunohistochemistry and confocal microscopy**

For immunohistochemistry (IHC), on the assigned day of sacrifice, animals were anesthetized by placing the rat in an induction chamber preloaded with isoflurane (5 % in oxygen). After induction, 2 % isoflurane was used for maintenance during PBS intracardiac perfusion with 50 mL of phosphate buffered saline (PBS, pH 7.2) The rats were then sacrificed by rapid decapitation, the brains quickly extracted and placed in ice cold ACSF. The brain‐stem and forebrain were dissected and frozen separately in optimal cutting temperature (OCT) and stored at −80°C until cryostat slicing.

The embedded forebrain and brainstem blocks were sectioned in a cryostat at 10 μm thickness, and thaw mounted on glass slides. Slides containing the neuroanatomically identified DVC and CeA regions were first fixed in 100 % cold methanol for five minutes, then briefly rinsed in PBS three times, five minutes each. Sections were then blocked and permeabilized with PBS containing 2 % bovine serum albumin (BSA) and 0.1 % Triton X-100 (Sigma-Aldrich, St. Louis, MO, USA) for one hour. Afterwards, brain sections were incubated with the primary antibody (see below) overnight at 4°C. Then slides were washed and incubated for two hours at room temperature in the dark with the secondary antibody (see below). Finally, slides were washed, mounted with Fluorsave™ (Calbiochem, San Diego, CA, USA) and stored at 4°C in the dark. Staining controls were performed by incubating with PBS instead of the primary antibody or both antibodies (data not shown).

In order to visualize neurons, mouse monoclonal anti-NeuN (Millipore, Billerica, MA, USA; dilution 1:1000 in PBS-BSA) was used. Alexa-555 goat anti-mouse immunoglobulin G (IgG) (Jackson ImmunoResearch Laboratories, West Grove, PA, USA; dilution 1:1000 in PBS-BSA) was used as the secondary antibody to detect anti-NeuN staining. TNF-α protein was visualized with polyclonal rabbit anti-TNF-α (eBioscience, Glostrup, Denmark; dilution 1:100 in PBS-BSA) and Alexa-488 goat anti-rabbit IgG (Jackson; dilution 1: 1000 in PBS-BSA). Mouse monoclonal anti-rat endothelial cell antigen-1 (RECA-1) (Genway Biotechnology, San Diego, CA, USA diluted 1:10 in PBS-BSA) was used as the primary antibody to localize blood vessels [52]. RECA-1 staining identifies terminal arterioles, arterial capillaries and venous capillaries, postcapillary-sized venules, and collecting venuoles [53,54]. In the hippocampus, arterial microvessels and capillaries were shown to stain more strongly then venous vessels. While there are differences at the anatomic and molecular levels between microvessels [55], they were not subclassified in this study. Alexa-555 donkey anti-mouse IgG (Invitrogen; dilution 1:1000 in PBS-BSA) or Alexa-633 donkey anti-mouse (Invitrogen; dilution 1:1000 in PBS-BSA) was used as the secondary antibody to detect anti-RECA-1 staining. To detect the presence of intercellular adhesion molecule 1 (ICAM-1), rabbit anti-rat CD54 monoclonal antibody (Millipore; dilution 1:100 in PBS-BSA) was used with Alexa-488 donkey anti-rabbit IgG (Invitrogen; 1:1000 in PBS-BSA). Finally, for nuclei staining, the slides were washed with PBS, and then incubated with 5 μg/ml 4′-6-diamidino-2-phenylindole (DAPI) (Sigma-Aldrich) at room temperature for five minutes.

### **Confocal microscopy**

Confocal microscopy (Zeiss 510 Meta, Göttingen, Germany) was performed with an x63 objective lens (oil, numeric opening 1.4). We used an argon laser (excitation 488, emission 505 to 530 nm) for Alexa-488, a helium laser (excitation 543, emission 585 to 615 nm) for Texas Red and a krypton-argon laser (excitation 647 nm, emission 660 to 700 nm) for Alexa-647. Images were collected sequentially to avoid cross-contamination between fluorochromes. A series of 15 optical sections was projected onto a single image plane and scanned at 1024 × 1024 pixel resolution. Images are pseudocolored for visualization.

## **Results**

### ***Ccl2*,*NOS-2*,*TNF-α*,*Tnfrsf1a*and*CD74*gene expression during alcohol withdrawal**

The objective of this study was to identify changes in innate immunity in the CNS during the first 48 hours of alcohol withdrawal following a single long-term exposure in two regions anatomically associated with the emotional and autonomic instability associated with alcohol withdrawal. We examined the gene expression of Ccl2, NOS-2, TNF-α, Tnfrsf1a and CD74 as surrogate markers of inflammation in the CeA of adult male rats exposed to a liquid alcohol diet for thirty-five days and in similarly treated animals four hours and forty-eight hours into alcohol withdrawal. The CeA is an alcohol-responsive part of the limbic system, thought to play a major role in drug addiction and the coordination of autonomic and emotional behaviors [28]. We took additional confirmatory measures in a second directly connected brain region [33], the alcohol responsive DVC, also relevant to physiologic sympathetic response during alcohol withdrawal [31].

**Figure 2:**
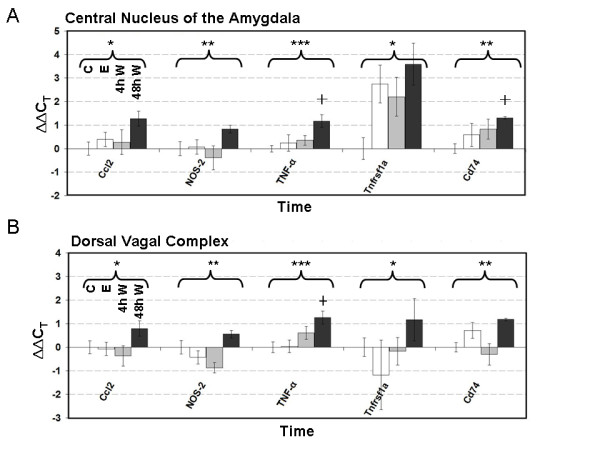
**qRT-PCR analysis of expression of*Ccl2*,*NOS-2*,*TNF-α*,*Tnfrsf1a*and*CD74*during the first 48 hours of alcohol withdrawal.** Alcohol-treated rats were fed the Lieber-DeCarli liquid alcohol diet for 35 days prior to forced withdrawal. qRT-PCR was performed on CeA and DVC samples. All transcripts were found to have a significant treatment effect by two-factor ANOVA, and to change significantly as a result of alcohol treatment and withdrawal. **(A)** CeA mean ∆∆C_T_ values for the transcripts *Ccl2*, *NOS-2*, *TNF-α* and *Tnfrsf1a* and *CD74* in control (C; N = 10), chronically alcohol exposed (E; N = 5), and 4 hours (4 h W; N = 5) and 48 hours (48 h W, N = 3) withdrawn rats. **(B)** DVC mean ∆∆ C_T_ values (C N = 11; E = 5, 4 h W n = 7, 48 h N = 5). A ∆∆C_T_ = 1 is a doubling of control mRNA levels. Error bars represent the +/−SEM. *Significant treatment effect identified via two-way ANOVA (*P* ≤ 0.05); **Significant treatment effect identified via two-way ANOVA (*P* ≤ 0.01); ***Significant treatment effect identified via two-way ANOVA (*P* ≤ 0.005); † Significant post hoc Tukey’s test versus control, (*P* ≤ 0.01.) ANOVA, two-way analysis of variance; *Ccl2*, chemokine (C-C motif) ligand 12; CeA, central nucleus of the amygdala; DVC, dorsal vagal complex; *NOS-2*, inducible nitric oxide synthase; qRT-PCR, quantitative reverse transcription polymerase chain reaction.

*TNF-α* (*P* = 2.85 x10^-4^), *NOS-2* (*P* = 0.005), *Ccl2* (*MCP-1*, *P* = 0.041), MHC II invariant chain *CD74* (*P* = 0.007), and the TNF receptor *Tnfrsf1a* (*P* = 0.041) all showed statistically significant cumulative treatment effects identified by analysis of variance (ANOVA), as shown in Figure 2. While we also noted significant regional expression differences in *TNF-α* (*P* = 7.32 × 10^-4^) and *Ccl2* (*P* = 1.73 × 10^-5^), there were no significant region-treatment interactions. Figure 2A shows the increase in CeA mRNA levels of *TNF-α*, *NOS-2*, *Ccl2* and *CD74* during withdrawal. Further examination shows that these changes are largely attributable to increases measured at 48 hours, reaching a near doubling (∆∆C_T_ = 1) of control levels, while chronic alcohol exposure resulted in relatively small increases in expression. Follow-up pair-wise post hoc Tukey’s HSD testing to identify differences between individual time points was then performed to compare individual treatment conditions to one another. Time-point-specific testing showed that at 48 hours changes in both *TNF-α* and *CD74* mRNA were significantly different from control values by post hoc Tukey’s testing (*P* ≤0.05). *Tnfrsf1a* expression in the CeA was also affected by alcohol exposure and withdrawal, though its pattern was distinct from the other transcripts. The TNF-α receptor mRNA was strongly upregulated during chronic alcohol exposure, with a nonsignificant trend toward an additional increase during withdrawal.

Figure 2B shows the analogous gene expression in the DVC. Again for *Ccl2*, *NOS-2*, *TNF-α*, *Tnfrsf1a* and *CD74*, we saw a significant mRNA induction over the first 48 hours of withdrawal reaching an approximate doubling of control mRNA levels. As in the CeA, Tukey’s testing identified a significant difference between 48-hour *TNF-α* levels in comparison to control (*P* < 0.01). Additionally, a statistically significant difference was found in the DVC expression levels of *CD74* between four and forty-eight hours of withdrawal (*P* ≤0.05). Similar to the chronic changes seen in the CeA, DVC samples taken during prolonged exposure also showed relatively small changes in mRNA levels of *Ccl2*, *NOS-2*, *TNF-α* and *CD74*. However, DVC levels of these transcripts were mildly reduced rather than upregulated in chronically exposed animals, though these effects were nonsignificant by post hoc testing. Again, *Tnfrs1a* showed a unique expression pattern, with a large average decrease during chronic exposure.

### **Localization of TNF-α, RECA-1 and ICAM-1 expression**

**Figure 3:**
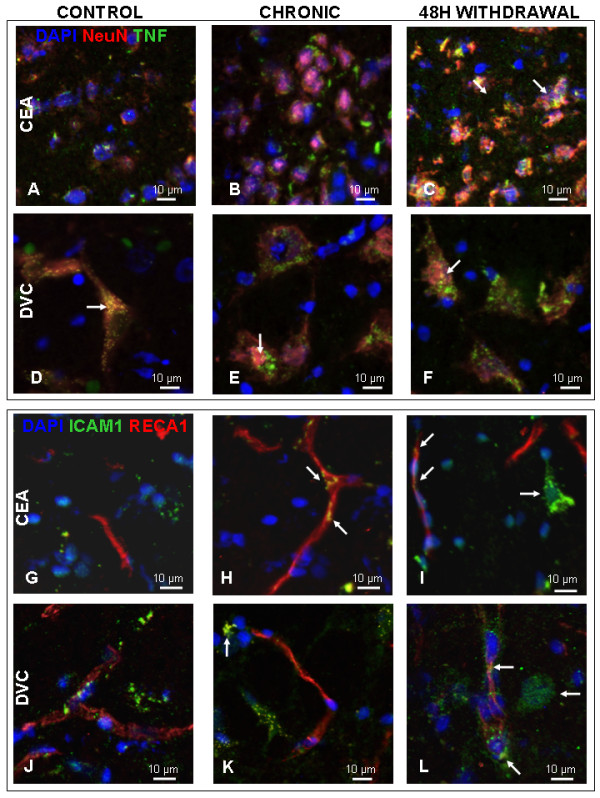
**Immunohistochemical evaluation of the alcohol-induced inflammatory response. (A** to **F).** TNF-α reactivity in the neural compartment in the CeA **(A** to **C)** and DVC **(D** to **F)** in control animals (**A,** **D;** N = 4) and following chronic alcohol exposure (**B,** **E;** N = 2) and 48 hours into withdrawal (**C, F;** N = 2). Neurons are stained red with neuronal nuclear antigen (NeuN) and nuclei with DAPI shown in blue. The arrows in panels **C.**, **E.** and **F.** show the expression of TNF-α (green) surrounding cells expressing the neural marker NeuN. Dual labeling of a cell for both NeuN and TNF is shown in yellow. **(G** to **L).** ICAM-1 reactivity (green) in the endothelial compartment in the CeA **(G** to **I)** and DVC **(J** to **L)**, following chronic alcohol exposure **(H,K)** and 48 hours into withdrawal **(I,L)**. Endothelia are stained red with RECA-1 and nuclei with DAPI shown in blue. The arrow in panel **H** shows the expression of ICAM-1 by cells also expressing the endothelial cell marker RECA-1. Panels **I** and **L** have arrows showing small cells with a high degree of ICAM-1 staining found commonly in the chronic and withdrawal tissue that are absent in the control condition. Dual labeling of a cell for both RECA-1 and ICAM-1 is shown in yellow. CeA, central nucleus of the amygdale; DAPI, 5 μg/ml 4′-6-diamidino-2-phenylindole; DVC, dorsal vagal complex; ICAM‐1, intercellular adhesion molecule 1;NeuN, neuronal nuclear antigen; RECA‐1, rat endothelial cell antigen‐1.

As TNF-α is known to be a central provocateur of inflammation, we sought to confirm the presence of the TNF-α protein in alcohol-withdrawn animals as a marker of active early inflammation, and to compare IHC staining of CeA in the control condition, and in similarly treated animals with an eight-month chronic alcohol exposure and forty-eight-hour withdrawal. In this way, the IHC images serve as a qualitative confirmation of the presence of an innate immune response during alcohol withdrawal as suggested by our quantitative PCR measures. Representative images of the staining in these three conditions are shown in Figure 3A to C. Consistent with the normal noninflamed state of the brain, the control CeA samples showed only sparse TNF-α staining. Samples taken during chronic alcohol exposure showed more pronounced TNF-α staining, demonstrating persistent elevations after an eight-month exposure, consistent with our qPCR measures. CeA 48-hour withdrawal samples showed increased TNF-α staining in comparison to both control and chronic samples, confirming the presence of a protein-level inflammatory response. Additionally, CeA withdrawal samples showed dual labeling of TNF-α and NeuN, suggesting neuronal production of the cytokine. This differs from the control and chronic conditions where CeA TNF-α staining appears to be localized to discrete areas surrounding cells expressing the neural marker NeuN, as shown in Figure 3B to C. Similar, but less pronounced findings were observed in the DVC, with increased TNF-α staining in the chronic and withdrawal conditions. However, no localization differences were observed in the DVC (Figure 3D to F), and intracellular TNF-α staining can be seen in all treatments, including in the control DVC neuron in Figure 3D.

To determine if neurovascular endothelial cells undergo a response consistent with inflammatory activation, we performed IHC for ICAM-1 (CD54), a cellular adhesion molecule upregulated by TNF-α [56-58]. Control CeA samples showed limited RECA-1 endothelial staining with no ICAM-1 colabeling, consistent with normal vascular endothelia (Figure 3G). However, following the eight-month alcohol exposure, RECA-1 and ICAM-1 colabeling became apparent (Figure 3H). This colabeling was also present in the 48-hour withdrawal condition, confirming the presence of endothelial cells expressing cellular adhesion molecules during withdrawal. Again, these findings were confirmed in the DVC as shown in Figure 3K to L. Finally, during exposure and withdrawal there is considerable ICAM-1 expression in the cells surrounding the vasculature in both the CeA and DVC (Figure 3H to I, K to L).

## **Discussion**

This study demonstrates *in vivo* that alcohol withdrawal following a single long-term alcohol exposure is associated with increased inflammation. We confirmed this observation in two brain regions neuroanatomically associated with emotional and autonomic regulation that are notably disrupted during withdrawal: the central nucleus of the amygdala (CeA) and the dorsal vagal complex (DVC). Our study demonstrates the increased expression of mRNAs specific for several inflammatory markers in these regions including the inflammatory cytokine *TNF-α* and its receptor *Tnfrsf1a*, monocyte chemoattractant *Ccl2*, inducible nitric oxide synthase *NOS-2*, and the major histocompatability complex class II antigen (MHC II) invariant chain *CD74* over a 48-hour withdrawal. By demonstrating IHC staining for TNF-α surrounding NeuN-positive neurons and ICAM-1 in RECA-1-expressing endothelia, we also verified the presence of an inflammatory response at the protein level. Additionally, consistent with other previously reported studies, we observed a mild inflammatory state associated with chronic intake. These qRT-PCR and IHC studies indicate that the first 48 hours of alcohol withdrawal are characterized by an exacerbation of alcohol-induced proinflammatory changes in brain regions anatomically associated with the maintenance of emotional and cardiorespiratory homeostasis which are known to be disrupted during withdrawal in humans [24,59] and animal models [27,31] over the same time frame.

The protein products of the TNF-α and Tnfrsf1a genes act together to activate and amplify the inflammatory response, and increased expression during alcohol withdrawal suggests that these processes are activated. NOS-2 encodes inducible nitric oxide synthase, suggestive of concurrent oxidative stress. The concomitant increases in CD74 and Ccl2 may indicate that changes in immune cell composition are occurring, potentially altering the number, activation state or type of antigen-presenting cells in these nuclei during withdrawal. These findings may have important consequences on emotional and cardiorespiratory regulation, as CNS inflammation has been shown to alter physiology and behavior. For example, injection of TNF-α into the ventricles causes elevations in mean arterial pressure [60] and repeated microinjections of TNF-α and Ccl2 into the amygdala prior to prolonged alcohol exposure have been associated with an exaggerated anxiety-like response during withdrawal [22,61]. Similarly, peripheral lipopolysaccharide (LPS) injection in rats has also been shown to amplify anxiety-like withdrawal behavior [22]. Conversely, TLR4 −/− knockout mice that fail to express a receptor critical to the innate immune response have been shown to be resistant to behavioral and cognitive changes associated with alcohol exposure. Specifically, TLR4 −/− knockout mice show neither the decreased exploratory activity eight hours into withdrawal following a five-month alcohol exposure nor the impaired cognitive testing following a fifteen-day withdrawal that is typical in the wild-type [62]. This suggests that these changes are dependent on an intact innate inflammatory response. Our studies support this conclusion by directly measuring increases in several innate immune signals within the first 48 hours of withdrawal, demonstrating an innate immune response as a primary element of withdrawal pathology. Consequently, repeated cycles of inflammation induced by CeA TNF-α microinjection as in the Knapp Study [22], or synthesized endogenously as a consequence of repeated episodes of exposure and withdrawal, may worsen withdrawal symptoms by amplifying this innate immune response.

Notably, inflammation in the CeA and DVC, brain regions that act to regulate emotional and physiologic homeostasis, could form a considerable barrier to abstinence and contribute to the cycle of negative reinforcement that sustains dependent drinking behaviors [63]. The therapeutic implications of this finding are important, suggesting that immunomodulators may be effective in treating emotional and autonomic dysregulation during withdrawal. Additionally, the involvement of embryologically, neuroanatomically and functionally diverse DVC and CeA brain regions raises questions of the generalizability of this active innate immune response in other brain regions. Further, the demonstration of a short-term innate immune response during withdrawal raises questions about the relationship between alcohol-related neurodegeneration and withdrawal rather than consumption in isolation. However, studies that confirm the presence of a sustained inflammatory response in these and other brain regions classically associated with alcohol-related neurodegeneration including the cerebellum, hippocampus, entorhinal and perirhinal cortices [34,35,64-66] along with direct assessments of neural injury comparing prolonged exposure with and without withdrawal periods are necessary, to further explore this hypothesis.

Earlier work has focused on the inflammatory consequences of long-term alcohol exposure, yet few studies have characterized these processes in withdrawal directly. Studies of neurons and glia exposed to alcohol in culture have shown a variable response, including increases in the generation of reactive oxygen species, prostaglandins [14], and NFkB DNA-binding [21] that has been purported to be both injurious to and protective of central nervous system cells [67]. *In vivo* studies have worked to clarify these seemingly contradictory findings, and suggest that these consequences are largely proinflammatory and injurious; following long-term alcohol exposure, murine frontal cortex samples have increased expression of NOS-2, ionized calcium binding adaptor molecule 1 (Iba1), and 3-nitrotyrosine protein adduct levels consistent with tissue injury [68], and demonstrate sustained increases in the production of TNF-α, MCP-1 and Il-1β in the brain following intraperitoneal LPS injection without direct CNS injury, confirming a proinflammatory state [4]. While direct studies of withdrawal are limited, Brown and colleagues showed that in hippocampal-entorhinal cortical slice cultures, repeated cycles of exposure and withdrawal led to increased neural damage that could be partially inhibited by treatment with the PLA2-inhibitor mepacrine and the anti-inflammatory lipid docosahexaenoic acid [35]. In contrast, a single four-day exposure of alcohol and twenty-four-hour withdrawal period was not associated with increased TNF-α, IFN-Y, Il-1b, Il-4, Il-5, Il-13 or Cxcl1 protein levels in the brain of rats [69]. Here, our measures suggest that a single long-term alcohol exposure followed by a 48-hour withdrawal is sufficient to induce a significant central nervous system inflammatory response, larger in magnitude than that associated with chronic exposure. Other work from our laboratory examining early withdrawal transcription dynamics in the DVC [31] and CeA (in review) showed surprisingly large and extensive transcriptional responses suggestive of profound changes in intracellular signaling, potentially consistent with active inflammation. Thus, this study aims to follow up these results with the focus on neuroimmune processes. Its results, most notably the increases in *TNF-α*, strengthened by concomitant increases in the mRNA expression of *Ccl2NOS-2Tnfrsf1a* and *CD74*, provide more direct evidence of a neuroimmune response in these nuclei anatomically related to withdrawal’s emotional and homeostatic imbalance.

## **Conclusion**

In summary, the current findings show that alcohol withdrawal induces an acute exacerbation of the limited proinflammatory response seen during long-term chronic alcohol exposure at both the mRNA and protein level at 48 hours. Our *in vivo* measurement of increases in *TNF-α*, *Tnfrsf1a*, *Ccl2*, *NOS-2* and *CD74* during withdrawal suggests that the period can be viewed as an acute-on-chronic inflammatory process, where proinflammatory changes that occur during chronic alcohol exposure are worsened immediately following the removal of alcohol from the cellular environment. As a consequence of the roles of these regions in emotional and physiologic regulation, these findings may have important implications for the treatment of withdrawal-related autonomic and emotional dysfunction. Targeted investigations aimed at characterizing cell-specific interactions in neurons and glia, as well as studies aimed at pharmacologic manipulation of these central inflammatory pathways in these homeostatic brain regions may increase our understanding of withdrawal and its associated affective and cardiorespiratory effects.

## **Abbreviations**

ANOVA, two-way analysis of variance; BSA, bovine serum albumin; Ccl2, chemokine (C-C motif) ligand 2; CeA, central nucleus of the amygdala; COX-2, cyclooxygenase-2; CNS, central nervous system; DAPI, 5 μg/ml 4′-6-diamidino-2-phenylindole; DVC, dorsal vagal complex; Iba-1, ionized calcium binding adaptor molecule 1; ICAM-1, intercellular adhesion molecule 1; Ig, immunoglobulin; IHC, immunohistochemistry; IL, interleukin; MCP-1, monocyte chemotactic protein-1; HSD, honestly significant difference; LPS, lipopolysaccharide; MCH-II, major histocompatability complex class II antigen; NeuN, neuronal nuclear antigen; NOS-2, inducible nitric oxide synthase; OCT, optimal cutting temperature; PBS, phosphate buffered saline; qRT-PCR, quantitative reverse transcription polymerase chain reaction; RECA-1, rat endothelial cell antigen-1; TNF-α, tumor necrosis factor-alpha.

## **Competing interests**

The authors declare that they have no competing interests.

## **Authors’ contributions**

KF conceived of the study, carried out the qRT-PCR, performed the statistical analysis and drafted the manuscript. AB participated in the experimental design and carried out the qRT-PCR and IHC studies with MMB. RV directed the data normalization and statistical analysis. MMS participated in the experimental design, and performed the animal experiments for the qRT-PCR. GEG, JBH, DCH and JSS contributed specific subject matter expertise in molecular biology, animal models of alcohol exposure, neuroscience and neuroinflammation. All authors read and approved the final manuscript.
